# Interdisciplinary Lessons Learned While Researching Fake News

**DOI:** 10.3389/fpsyg.2020.537612

**Published:** 2020-12-16

**Authors:** Char Sample, Michael J. Jensen, Keith Scott, John McAlaney, Steve Fitchpatrick, Amanda Brockinton, David Ormrod, Amy Ormrod

**Affiliations:** ^1^Idaho National Laboratory, Idaho Falls, ID, United States; ^2^Institute for Governance and Policy Analysis, University of Canberra, Canberra, ACT, Australia; ^3^Department of Linguistics, De Montfort University, Leicester, United Kingdom; ^4^Department of Psychology, Bournemouth University, Bournemouth, United Kingdom; ^5^Terra Schwartz, Canberra, ACT, Australia

**Keywords:** fake news, discipline, behaviors, values, rhetoric, politics, deception

## Abstract

The misleading and propagandistic tendencies in American news reporting have been a part of public discussion from its earliest days as a republic (Innis, 2007; Sheppard, 2007). “Fake news” is hardly new (McKernon, 1925), and the term has been applied to a variety of distinct phenomenon ranging from satire to news, which one may find disagreeable (Jankowski, 2018; Tandoc et al., 2018). However, this problem has become increasingly acute in recent years with the Macquarie Dictionary declaring “fake news” the word of the year in 2016 (Lavoipierre, 2017). The international recognition of fake news as a problem (Pomerantsev and Weiss, 2014; Applebaum and Lucas, 2016) has led to a number of initiatives to mitigate perceived causes, with varying levels of success (Flanagin and Metzger, 2014; Horne and Adali, 2017; Sample et al., 2018). The inability to create a holistic solution continues to stymie researchers and vested parties. A significant contributor to the problem is the interdisciplinary nature of digital deception. While technology enables the rapid and wide dissemination of digitally deceptive data, the design and consumption of data rely on a mixture of psychology, sociology, political science, economics, linguistics, marketing, and fine arts. The authors for this effort discuss deception’s history, both old and new, from an interdisciplinary viewpoint and then proceed to discuss how various disciplines contribute to aiding in the detection and countering of fake news narratives. A discussion of various fake news types (printed, staged events, altered photographs, and deep fakes) ensues with the various technologies being used to identify these; the shortcomings of those technologies and finally the insights offered by the other disciplines can be incorporated to improve outcomes. A three-point evaluation model that focuses on contextual data evaluation, pattern spread, and archival analysis of both the author and publication archives is introduced. While the model put forth cannot determine fact from fiction, the ability to measure distance from fact across various domains provides a starting point for evaluating the veracity of a new story.

“If it is not true, it is very well invented.” —Giordano Bruno

## Introduction

Fake news has a long history in America (McKernon, 1925; Innis, 2007), becoming internationally recognized as a problem in 2016, the year it was declared word of the year by Macquarie Dictionary (Lavoipierre, 2017). The re-emergence of the term “fake news” (Meza, 2017) served as an inflection point for academics across various disciplines. Some academics observed the commonalities between “fake news” and propaganda that uses a different delivery mechanism (Younger, 2018), whereas others observed greater sophistication, customization, and weaponization (Younger, 2018; Verrall and Mason, 2019). Those in academia and government who recognized this threat, some as early as Szfranski (1997), others soon after (Cybenko et al., 2002). Szfranski (1997) suggested that the weaponization of deceptive information would require protection of both combatants and non-combatants alike. While some groups of people are more resilient against deceptive data (Bjola and Papadakis, 2020) suggesting a cultural component, significant populations remain vulnerable. The vulnerable also includes journalists, who repeat the stories that align with their own values. Even the journalists’ verification and validation mechanisms are corrupted by algorithms that provide information that aligns with targeted beliefs. Indeed, according to some behavioral scientists, all are vulnerable to the messages that confirm biases (Oswald and Grosjean, 2004).

Media spheres such as journalism require their journalists to act as watchdogs of information-sharing for its global citizens. Their position in the world holds responsibility to provide independent truth and legitimacy to its audience by providing fact-checking. General verification procedures will be covered to transition into discussion about the importance of interdisciplinary work. Journalists intercept deception by reporting on the truth of our reality, having a general agreed normative approach to fact-checking including combating fake news narratives even if interpreting false claims is still very much subjective (Graves, 2018; Mena, 2019). Five elements of fact checking provided by Bratich (2020) include choosing claims to check, contacting the source of the claim, tracing false claims, working with experts, and showing their (journalists) work. Within this frame, journalism has become loaded with uncertainty, mistrust, and manipulation from its user engagement and many other trends, such as politics and emerging technologies, which intersect it (Waisbord, 2018).

Pomerantsev and Weiss (2014) identified five goals of disinformation, of which fake news is a subset, these goals include paralysis, demoralization, confusion, blackmail, and subversion. Disinformation campaigns will seek any and all of these goals each of the five goals represents a strategy to use against a targeted group of people. For example, credible news stories that report opposite stories on the same event can, if both are professionally done, confuse a person who is new to the story and environment, rather than simply sway the person to one side or another.

### Fake News, Disinformation, and Manipulation

The use of disinformation and misinformation in news has a long history. Some scholars have focused on misinformation, the inadvertent release of misleading or factually incorrect information, or disinformation, involving the intentional diffusion of factually incorrect claims for political purposes (Bakir and McStay, 2018; Bennett and Livingston, 2018). From a perspective of information warfare, however, claims need not be false to have strategic value in manipulating an audience (Schafer, 2018). Well-timed, factually correct information can be as effective as a lie; when this occurs, the information becomes weaponized. This section develops the distinction between information and its weaponization as the movement from an information logic to an identity logic within communications.

Information is classically defined as “current data about developments in and the status of” a system at any given time (Downs, 1957, p. 79). An information logic has three components. First, there is a temporal dimension. As information refers to the current status of a system or scene of events, it has a fleeting duration, “information that is repeated is no longer information. It retains its meaning in the repetition but loses its value as information” because it no longer updates one’s understanding of a state of affairs (Luhmann, 1995, p. 67; Lash, 2002). The same materials may resurface repeatedly, but this defines an extended present that sublimates past and future and is no longer operating in an informational mode (Rushkoff, 2013).

Second, the contextualized component includes the scene or situation in which activity takes place. Contextualized data are bound to the environment where created or resides. In this sense, data, particularly digital data, when taken out of context, perturb the environment, and traces from the perturbation will remain.

Information has a third characteristic: information descriptive, rather than moralizing or hortatory, does not call for action (Burke, 1969, p. 41). Thus, information contrasts with emotionally polarizing communication content. Whereas the experience of sensory data may provide an updated status about local states of affairs, emotions “do not give us any information about the world” (Wittgenstein, 1967, p. 491).

Both misinformation and disinformation point to informational disorders, an enduring aspect of the Downsean tradition within political science. Accurate information about states of affairs within a political system has remained a critical currency, often in too short supply, which citizens require to make informed democratic choices (Converse, 1962; Grofman, 1995; Delli et al., 1997; McGann, 2006). Fake news is often taken as a species of disinformation as it is both a fabrication and “mimics news media content in form but not in process or intent” (Lazer, 2018, p. 1094). From this perspective, misinformation and disinformation are distorting as these phenomena provide errant premises on which to make decisions in light of pregiven preferences (Hochschild and Katherine, 2015; Mathiesen, 2018). And, while deception might be a short-term strategy for elected officials, if they seek reelection, there can be costs to deception during elections (Ferejohn, 1990, p. 10). More recently, the rise of polarized electorates makes possible the continuation of informational disorders.

### Preparing for Fake News

Deception is not limited to the political realm. Health/medicine (Springer, 2020), finance (Cybenko et al., 2002), the military, and cyber domains are a few of the other environments where deceptive data or fake news have successfully been deployed. Deception relies on tricking both sensation and perception. In order for information to be perceived the data must first be sensed. Human perception and information processing are still not fully understood (De Faveri et al., 2017).

“Persuasion lies at the heart of political communications” (Flanagin and Metzger, 2014, p. 1). In order for fake news to be effective, the deception must first be formed then communicated. The communication process where sender and receiver share the same perception of information was defined by Gerbner (1956). Gerbner’s model of communications identifies the role of perception and contextualization in the message creation phase. Successful deception relies on influencing the thinking of the target (De Faveri et al., 2017). The target in the case of fake news is the human mind, and journalists are humans and thus vulnerable to disinformation.

Because the target is the human mind, a brief discussion on factors that feed into decision-making is relevant. Before information processing can occur, the data that form the information must first be sensed and then perceived. Sensing occurs when one or more of the five senses are stimulated. Generally speaking, sight and sound predominate in sensory stimulation particularly, so in the online world where fake news prevails, sensory deprivation makes possible the ability to control perception. Perception provides the input into decision-making, so a lack of stimuli (sensation data) or manipulated sensation data designed as context triggers the initial unconscious, neural response for specific actions (Gazzaniga, 2014). Conversely, an un-sensed event is never perceived or a non-event.

While much focus on decision-making centers on motivation, the authors would be remiss if they did not list additional factors. Decisions rely on several factors that in addition to motivation include patience/impatience, risk attitude, and ambiguity attitude (Gazzaniga, 2014). These different factors suggest that the manner in which decisions are made is diverse. Any single factor or combination of factors and predicting which factors dominate in any decision are most accurate as a *post hoc* exercise. Furthermore, all factors discussed in the decision-making process are situationally dependent.

Decisions can be broken down into two types: conscious, otherwise known as action–outcome decisions, and unconscious, also referred to as stimulus–response (Dijksterhuis, 2004; Kahneman, 2011; Gazzaniga, 2014). Both types of decisions are influenced by biases. De Faveri et al. (2017) described patterned deviations from fact in perception as biases and heuristics—cognitive shortcuts (McAlaney and Benson, 2020). Regardless of the type of decision being made, the biases still influence. However, the message creation can vary.

De Faveri et al. (2017) examined deception in the hostile cyber environment and described three groups of biases: personal and cultural, organizational, and cognitive. It was noted that while highly effective, biases are difficult to obtain (ibid). However, this observation preceded widespread knowledge of the role of social media in fake news targeting and dissemination (Shu et al., 2017 several sources).

The new role of social media in content creation, delivery, and dissemination of fake news has changed the landscape in unanticipated ways, requiring a reexamination of ways to identify and ultimately mitigate this type of deception. For this reason, the authors are examining some of the common disciplines involved in fake news or digital deception within the model describing content, distribution, and archives defined by Sample et al. (2018).

### Deception as a Strategy

The assumption and assertion of a paper such as this are the role of fake news as a means to achieve political, social, and potentially other forms of influence utilizing deception as a strategy. Deception as strategy has roots in ancient human behavior, observed in the earliest histories including Greek mythology (Phaedrus, 2008, p. 438). In more recent times, Erfurth’s treatise on Surprise (Erfurth, 1943) provides a number of helpful insights. He observes that almost all decisive military victories have been preceded by surprise, which relies on secrecy and speed. Deception is a form of surprise, providing a means to unbalance an opponent through uncertainty. Handel’s detailed analysis of deception at the strategic and operational levels in World War II also offers key observations. Deception must be believable to the target audience, with sufficient resources and time invested in a coherent narrative to reinforce existing beliefs: “The susceptibility to conditioning is one of the most fundamental human proclivities to be exploited by deception operations” (Handel, 1987, p. 14). Conditioning greatly precedes the actual event of deception. Conditioning lays the groundwork upon which the deception capitalizes.

Disinformation, a critical form of and enabler of deception, has a history in both warfare and state security functions. The use of disinformation as a form of deception is examined by Whaley (2007, p. 8), in the historical context that it was originally a World War I term applied by the German General Staff and then adopted by the Russians. Applying Shannon’s communication model, relevant but false information is fed into a communication channel, forming a third transmission category to signal and noise. This third category described by Whaley (ibid) may be disinformation or misinformation depending on intent. Misinformation is inadvertent, whereas disinformation deliberately seeks to overload, discredit, or realign an audience’s information management capabilities. Given the requirement to consider intent, disinformation has little utility without a purpose. Having understood the target and obtaining a means to access information and information networks and then subsequently exploiting those networks to expand access, disinformation provides the means to utilize supporting conditioned biases and narratives with the intent to influence perceptions and behaviors (Waltz, 2008, p. 4).

The Soviet concept of *maskirovka* probably best encapsulates the complexity of the problem space surrounding deception. Although the term *maskirovka* can be defined as camouflage, it extends in Soviet doctrine across a broad array of strategic, operational, and tactical measures to obscure intent, maintain security, and confuse the adversary (Glantz, 2006, p. 2). While there are numerous instances of these maskirovka strategies being successfully employed throughout World War II, which have helped inform modern doctrines and techniques, it is also important to note that failures have occurred when maskirovka was employed hastily, poorly coordinated, enacted by personnel with inadequate training (ibid, p. 14), or conducted in a stereotyped or patterned manner (ibid, p. 10).

A critical observation of the maskirovka concept is the employment of a variety of techniques at all levels, in a planned and coordinated manner that also sought to embrace complexity with significant focus on aligning tactical outcomes with strategic intent. This appears to be a commonality with today’s use of fake news. The employment of various tactical and operational approaches to achieve a broad strategic intent allows for multiple target audiences to be engaged with sometimes conflicting narratives and thematic episodes. This tactical and operational flexibility could be regarded as dangerous and counterintuitive from the strategic perspective, but it provides freedom of maneuver across the information environment and the ability to leverage the complexity of the modern information environment to achieve specific outcomes efficiently and with speed. The ability to roam widely, engaging with numerous audiences, themes, and narratives, at speed, appears to be a force multiplier in the employment of disinformation and fake news through social media and online forums. Moreover, this approach capitalizes on a number of perceived failures by trusted agencies to apply their moral and ethical narratives consistently, meaning that conflicting narratives by fake news agencies can always be excused by way of pointing to inconsistencies by previously trusted establishments who have perceived conflicts of interest, often amplified by the same fake news outlets.

The identification of target variables and ability to fashion-specific messages at an individual level, refined based on their personal and cultural data, appear to support the contention that a single, cohesive narrative is not required in the modern world. Fake news agents, marketers, and political organizations are able to target specific individuals based on individual data collected from internet fingerprinting and social media. This target variable data can distinguish at an individual level likely biases, beliefs, and likely actions through personality profiles. The more traditional media, government, and military refer to target audiences in a different, less precise manner, based on broad narratives and a focus on broad beliefs and groupings with an assumption that these descriptions will lead to specific group behaviors. It appears from these differences and the rapid evolution of these technologies that disinformation campaigns have a distinct advantage in the modern information environment. In the instance where one can focus on the issues specific to each individual and fashion the message to alter behavior around those issues at a personal and granular level, it appears that the narrative can be delivered in a micro, targeted manner. The alternative appears to be the delivery of grand narratives and themes supported by “trusted” agencies that rely on their self-perception of impartiality, which is quickly a target for fake news agencies and those who are likely to benefit from distrust of alternatives to the fake news narratives. It remains to be seen if the employment of fact checking, controlled narratives, and traditional information operations approaches is sufficient for the information environment of the future, but the results to date are not particularly positive. Perhaps part of the problem is the inconsistency inherent in modern life—it is not inconceivable to act contrary to one’s beliefs based on more personal, pertinent matters, which are fleeting. That is a matter priests, theologians, and ethicists have grappled with since the dawn of organized religion. Personality and culture, discussed later within this article, are factors that are likely to contribute to these outcomes.

The modern context of disinformation as applicable to fake news extends from the fundamental concepts of deception as a strategy and some of the principles discussed above. Susceptibility to conditioning, bias, narrative, and the exploitation of information and social networks are all fundamental to the concept of fake news. These concepts will be discussed in more detail throughout this article.

## Background

The weaponization of information is enhanced in the digital world where communities are created not only through borders and national boundaries, but also by shared thoughts that include shared hopes and fears (Bennett, 2012). In the information age where decision-making, especially in Western-style democracies, carries great importance, the ability to control sensing and manipulate perception in online communities is extremely valuable.

If war is political, and politics inhibits a variety of attributes of war, modern politics is in many ways invested in the preparation for war, and our existing politics may even stem from and reproduce a set of relationships established through war (Virilio and Sylvère, 2008). The existence of nuclear deterrence as a variation of the “stability-instability paradox” may incentivize subkinetic forms of warfare, which can be harder to deter precisely because efforts to restore deterrence conventionally can risk nuclear escalation (Gartzke and Lindsay, 2019, p. 14). Clausewitz (1982) termed war as politics by other means. However, it is equally possible in the modern context of hybrid war, political warfare, gray-zone conflict, and the like; politics may be a continuation of warfare by other means (Foucault, 2003).

Information warfare weaponizes communications in order to effect change in a target audience in terms of their attitudes and behaviors. If content is the currency of propaganda, then timing performs a similar function for information weaponization. The strategic communication of information can be a “source multiplier” shaping one’s understanding of situations, as well as shaping “the operational environment” so as to neutralize an adversary, as well as advance one’s own strategic objectives (Armistead, 2004, p. 1). While information warfare retains the descriptor “information,” it denotes a field of communication that is transformative more than informative. Information operations achieve these ends by seeking to “influence, disrupt, corrupt, or usurp the decision making of adversaries” while protecting those capacities for one’s own side. Because decision making is largely biased, and biases are behavioral in nature, the shaping of attitudes and beliefs is key to success in this environment.

Although information warfare has often been conceptually confined to a space of military warfare, there is growing recognition that it “can take place in any situation across the spectrum of war or peace” (Morgan and Thompson, 2018, p. 10) whereby warfare extends into political life in a non-kinetic, non–physically violent form (Singer and Brooking, 2018, sec. Kindle: 325). This new type of warfare has been referred to as hybrid warfare (Commin and Filiol, 2013) and enacted in numerous countries (Atkinson, 2018), where fake news as a weapon of information warfare plays a prominent role (Younger, 2018).

Politics have become a problematic center in fake news reporting; journalists are criticized for being non-partisan during fact-checking procedures coupled by general misunderstandings that they are responsible for fact-checking future statements from politicians, leading to increased user distrust in mainstream media (Uscinski, 2015). These trends have caused a shift in the traditional hierarchical information sphere on how truth is reported, and interdisciplinary work has the opportunity to address some of these issues. Social media has transformed the landscape of information reporting, meaning that solutions to combat fake news depend on the flexibility of traditional journalistic pathways to produce fact-checking frameworks clear enough to account for such change. Much like cybersecurity issues faced today, the information flow of fake news is unprecedented and at times overwhelming; mounting pressures on journalists to discern truths has allowed for both increased verification and vulnerabilities to occur in reporting. For example, fact-checking the credibility of sources is a common task, but there are many examples in mainstream media where this is not the case, and many journalists recognize the presence of this information disorder (Plotkina et al., 2020). Information excess from virtual space produces a reporting experience that can cause fact-checking to be synonymous with instantaneous discernment of sources to then act, or not act, upon. Generally, journalists do agree that taking the time to do fact-checking thoroughly is more important than being the first to cover a story, but this is not always a tangible result (Schapals, 2018). Journalism, unlike many other spheres, have an advantage in its ability to report on evolving fake news concepts at the rate fake news articles are being produced because of its unique access to information streams.

There is an alternative view that modern information operations occur in a globally competitive environment, influenced by the integrated nature of the world trade and political systems. World war is unlikely due the looming threat of nuclear conflict, so instead political objectives are achieved through kinetic and non-kinetic proxy wars. Cyber and information are just some of the domains and environments where this competition plays out. Even from a warfighting perspective, in many cases the actual implementation of information operations as a component of military campaigning is focused on managing kinetic events against a narrative and coordinating non-kinetic effects to achieve specified outcomes. Therefore, the concept of information warfare can portray a more integrated and planned approach than is often the case. In reality, governments and societies, even totalitarian ones, must balance a variety of internal and external forces to shape their strategic objectives and narratives. For the purposes of this article, however, we will refer to information warfare in the context of ideologically and politically driven fake news, which seeks to manipulate, deceive, and change behavioral outcomes through disinformation for long-term strategic advantage.

While some aspects of campaigning fit within an informational economy, others bear a closer resemblance to the strategies and tactics of information warfare. Information by itself just informs an audience about the current state of affairs, leaving the parameters of decisions unchanged. Information becomes weaponized at the point that it shifts a target audience by either reshaping the environment or the preferences, attitudes, and even identities of the target audience in order to produce judgments, decisions, and behaviors favorable to the initiator (Marcellino et al., 2017, p. 9). This can be subtle. For example, rather than changing a person’s desire to vote for a particular party, it is enough to simply convince someone not to vote on the day of the election. Value sets and beliefs are not always the target. Sometimes it is enough just to influence behavior for a short period to achieve the desired outcome.

The purpose of news is to inform the target audience which differs from the weaponization of information that seeks to deceive or manipulate through transformation of one’s perception of a situation or through transformation of self-identity. This ties information operations to the rhetorical functions of communications of communication as information leaves things as they are, while rhetoric works on its subjects by influencing their identification with situations and their understanding of self-identity. The realignment of interests, attitudes, and beliefs through communications creates a “consubstantiality” between persons such that they come to see themselves as the same, at least within a certain set of parameters for acting (Burke, 1969, p. 21). Underlying the creation of consubstantiality involves shifting identifications with political objects and actors, as well as their understandings of themselves in relation to the political world. Identities are always relational, demarcating what one is and, simultaneously, what one is not (Connolly, 2002). Information warfare, therefore, involves the strategic and tactical use of information, which operates on the order of identities, shifting the alignments of a target from one set of political identifications to another, with the ultimate goal of shaping behavioral outcomes.

The focus on shaping identities and behavior is not limited to warfare between international adversaries. In contrast to the informational terrain of political conflict, which has informed models of spatial competition and political opinion formation, political preferences are not prior to campaigning but shaped by political identities, which are constructed through campaigning over time. Evidence points to the primacy of a politicized identity over information cues in understanding American political behavior. Political identity is irreducible to differences in issue positions as research shows policy positions even on fundamental issues such as abortion shift in line with partisan identities over time (Achen and Bartels, 2016; Mason, 2018). And while personality characteristics of candidates might be one calculation along with policy considerations, the explanation for political behavior predicated on the basis of an identity logic is quite distinct from the informational logic of policy preferences as policy preferences are derivative of partisan identifications rather than the other way around.^1^

An identity logic contrasts along all three dimensions of the informational logic. First, in terms of the target of definition, identities define actors rather than inform as to state of a situation in which action occurs. Personal identities are composed of “the commitments and identifications which provide the frame or horizon within which I can determine… what is good, or what ought to be done, or what I endorse or oppose” (Taylor, 1992, p. 27). The normative entailments of identities function in communications as an “inducement to action (or an attitude, attitude being an incipient act)” (Burke, 1969, p. 42).

Second, information and identity logics are temporally distinct. By overlooking the unique timestamps, deep fakes, computer-generated fake news, can work in deceiving targeted users. In contrast to the instantaneous and fleeting nature of information, identities temporally integrate an actor providing a sense of continuity over time and space (Miskimmon et al., 2014, p. 5). The repetition of identity claims perpetuates an identity narrative that preserves a sense of ontological security in the face of changing circumstances over time (Giddens, 1991, pp. 53–54). On the other hand, identities can be weaponized at the point that ontological security is put in jeopardy through communications that undermine one’s trust in political actors and institutions or one’s standing in the political system.

Third, in contrast to the descriptive nature of information, the moral horizons that define identities provides a language “for objects contain[ing] the emotional overtones which give us the cues as to how to act toward those objects” (Burke, 1984, p. 177). In online communications, “identity can be a shared feeling” as “people recognize themselves in the emotions of others” (Zaharna, 2018, p. 60). The contrasts to emotional appeals are descriptions of external objects and events without reference to the experience of those objects and events.^2^ Emotional appeals can problematize identities as in the case of repeated communications seeking to induce anger or fear can give rise to anxieties—a tactic used by the Russian social media efforts to move Americans to deidentify with the existing political order (Jensen, 2018).

### Fake News: Content Creation, Delivery, and Dissemination

Effective content creation relies on several different disciplines from target selection (military, political science, biology, psychology, and sociology) in service of creating memorable content. The content must resonate with the intended target, especially in an information-rich society. For this reason, a discussion of linguistics, psychology, and sociology is necessary. The delivery must be credible relying on psychology, sociology, linguistics, theater, and more recently data science. Dissemination often relies on technology, thereby introducing cybersecurity into the mix.

#### Linguistics: Analysis of Propaganda Tools

Linguistics is a discipline that is used in creation of deceptive data via rhetoric, but linguists are not consulted when countering efforts are necessary. Sample et al. (2018) cited the three well-known general attack types (ethos, pathos, and logos) as methods associated with supporting fake news and why these methods must be considered in any fake news countering solution. Of the three rhetorical groupings, each presents challenges as well as opportunities for automated processing by combining rules of linguistics and computer science when deploying computational linguistics.

The linguistic analysis of fake news must operate across a range of domains and disciplines, for every act of language is embedded in a context composed of a wide number of different influences and drivers (political, social, cultural, ideological… as well as the purely language-based). A study based on the identification of lexical and syntactic patterning as an identification and attribution tool (Conroy et al., 2015; Dey, 2018) can only be one step in the analysis. Similarly, a rhetorical analysis, detecting both individual figures of speech and appeals to ethos, logos, and pathos (see below), is vital, but can only be a single element in a much broader and deeper examination of the target texts. This article outlines a multidisciplinary approach for the identification and countering of fake news in general; we can also see how a blended, multidisciplinary methodology can operate at the linguistic and communicative levels. A helpful overview of one such blended approach is given by Zhou and Zafarani (2018), but in what follows, an outline model for the investigation of fake news is presented, based on analyzing it not as single acts of language, but as a *process* of targeted communication, consisting of several elements, all of which must be considered to permit a truly informed understanding of how fake news is *constructed*, and how it *functions*.

The following analytical schema is based on the seminal model of communication outlined by Shannon (1948), in which any communicative act is viewed as a process consisting of a *message* transmitted from a *sender* to a *receiver* via a *channel* (this is, of course, a simplification of the Shannon–Weaver model, but it is a useful starting point). The key thing to note here is that while we can study each element in isolation, a truly sophisticated analysis will consider how the various components interact and interrelate. For example, consider the issue of the chosen channel of transmission; different social media platforms operate in different ways (Twitter has restrictions of message length; Snapchat and Instagram are image-driven) and appeal to different demographics (Chen, 2020). Just as fake news must be carefully crafted to reach and appeal to specific target audiences, so any effective countering-strategy must consider the most appropriate communicative approaches and channels to mitigate against it. We know that there is a correlation between age of online users (and their political opinions) and their likelihood to retransmit fake news (Guess et al., 2019); work remains to be done on determining not just *why* this group is likely to fall prey to fake news, but in devising strategies for mitigating against this.

In order to conduct an effective analysis of fake news, we need to adopt the tools of corpus linguistics and establish a robust database (or *corpus*) of previous fake news campaigns. The examples cited in Tenove et al. (2018) and the two reports from the United Kingdom Information Commissioner’s Office. (2018a, b) provide a helpful starting point. This will permit the creation of a detailed taxonomy of fake news, looking at sender/receiver/channel and allowing a detailed analysis of types of message and their specific linguistic/rhetorical features. While work has already been done in this field (Digital Shadows, 2018; Molina et al., 2019), there is a pressing need for a much larger set of corpora, which will permit a fine-grained analysis.

The final area for future research is that of traffic and social network analysis; in order to truly understand how fake news functions, we need to examine the ways in which stories spread across a platform, and how various users, such as trolls, bots, and super users, act as prolific spreaders of misinformation. The development of tools for collecting, analyzing, and visualizing message spread over time is a priority. The issue of timescale is vital; we need to consider, for example, what factors drive a particular item of fake news to disseminate rapidly across information space, while counter-messages often lag far behind and over a much smaller area. One tool that offers a useful basis for R&D across the entire domain of social media platforms is FireAnt, a piece of open-source software devised by Lawrence Anthony and Claire Hardaker (Anthony, 2018), and another freely available tool is the OSoMe tool developed at Indiana University (OSoMe.). This allows the capturing of data from Twitter over a set timescale; the data can then be visualized as a social network map, permitting the identification of key nodes of transmission. This in turn allows a fine-grained analysis of message spread and the possibility of targeting any countering-strategy toward the most prolific transmitters. The challenge will be to devise tools that can operate across the whole range of social media platforms (the image-driven, multimedia-rich domains of Instagram and Snapchat will be particularly testing).

##### Ethos: define a person or group

“Persuasion lies at the heart of political communication” (Flanagin and Metzger, 2014, p. 1); thus, the role of the messenger is highly relevant. For this reason, the messengers are targeted, as mentioned above. Gu et al. (2017) observed that the cost to discredit a reporter was $50,000. Flanagin and Metzger (2014) noted the role of credibility in presentation, as well as perceptions of honesty and fairness, even when the message remained constant. Ethos applied to the messenger will be further discussed in the archival data subsection of this article.

Ethos defines the target or messenger (Cockcroft and Cockcroft, 2005, pp. 28–54). The definitions for this activity can be positive or negative. In some cases, popular personalities or celebrities endorse politicians or politics (Scott, 2006) or cures, and in other cases, negative nicknames are used to both of these topics associated with a considerable amount of fake news. In some cases, trusted reporters are targeted in an effort to damage their credibility. A second type of ethos involves the messenger and will be further discussed in the archive section. Attacking the messenger can take the form of discrediting a reporter, a publisher, an editor, or any other entity in the news supply chain.

Fake news will inevitably build on the ideological/cultural/political values of the intended audience, or it will fail. Not only must it “speak the audience’s language,” as it were, but it must also operate within the frame of reference held by that audience. In so doing, the “us” against “them” narrative that is commonly deployed can take hold adding an emotional tie-in. This emotional tie-in is further discussed in the pathos subsection.

When ethos is deployed, the good–bad dichotomy prevails, even though most individuals have both good and bad personality traits, nicknames suggesting malevolence or benevolence with the target but not both. The use of ethos appeals to tribal identification and behaviors. Thus, this form of propaganda can be used with better efficacy on homogeneous groups in societies where fear of new or other groups predominates over curiosity or hope (Hofstede et al., 2010). In these societies, “in groups” are viewed as having significantly different values than “out groups.”

##### Pathos: appeal to emotion

This rhetorical grouping is characterized by the appeal to emotions; thus, the emotions of fear (Montgomery, 2017) and hope (Menz, 2009) have a long-documented history of use in the political arena. Emotions play a critical role in political propaganda. In contrast to the descriptive nature of information, the moral horizons that define identities provide a language “for objects contain[ing] the emotional overtones, which give us the cues as to how to act toward those objects” (Burke, 1984, p. 177). In online communications, “identity can be a shared feeling” as “people recognize themselves in the emotions of others” (Zaharna, 2018, p. 60). The contrasts to emotional appeals are descriptions of external objects and events without reference to the experience of those objects and events.^3^ Emotional appeals can problematize identities just as in the case of repeated communications seeking to induce anger or fear can give rise to anxieties—a tactic used by the Russian social media efforts to move Americans to deidentify with the existing political order (Jensen, 2018). Fear and hope have also been used in as motivators in military matters.

Of the three approaches, pathos is the most immediately effective, acting as a means of short-circuiting logic and rational thought and aiming to evoke an immediate emotional response. This is due to the emotional nature of decision-making. When appealing to pathos, punctuation can be a valuable tool. Punctuation was shown to act as a marker for propaganda in Israeli political discourse (Shukrun-Nagar, 2009).

##### Logos: appeal to logic

Any logos-based approach is highly challenging. Human beings are driven primarily by emotions, and the use of logical reasoning and data-driven persuasion will founder on the lack of statistical and general mathematical knowledge in the general public. Of course, statistics can be easily manipulated to customize narratives. In some cases, as noted by Pomerantsev and Weiss (2014), any narrative can be created, and a supporting reality can be created to support that narrative.

Partial truths and decontextualized facts. This type of logic is sometimes effective in political and scientific arenas. Consider the antivaccination movement that believed that vaccinations caused autism (Gross, 2009). An unexplained rise in the number of autism cases along with a discredited article from a scientist (Wakefield, 1999) with celebrity endorsements (Antrim, 2018) sounding credible created a combination of an ethos- and logos-based appeal. Many conspiracy theories contain elements of logos mixed with pathos. The messaging must include terms and resonate with the target audience; word choices should reflect similar or the same words that the targeted group would use.

#### Psychology: Understanding Individual Behaviors and Thoughts

Humans are fundamentally social creatures. Our social worlds are complex and require us to sift through information to determine what is truthful and what we need to know to maximize our survival and personal growth. However, the amount of information that we process is extensive and, in our digital age, delivered to us at high intensity. As noted previously, heuristics are strategies that we use to take cognitive shortcuts in order to handle this information overload (Tversky and Kahneman, 1983) and are also known as cognitive biases. On occasion, these biases may lead us to come to the wrong conclusion or to take the wrong action, but ultimately such biases are an adaptive strategy that helps us navigate our environment. Nevertheless, these biases may be targeted and exploited by the authors of fake news. For example, the secrecy heuristics may lead people to believe that any information that is presented as being from a secret source is more reliable (Travers et al., 2014). This heuristic is exploited in fake news stories that proclaim to reveal “leaked” information. The acceptance of fake news can be further increased through the use of images to accompany the narrative that is being put forward (Newman et al., 2012), which again may exploit biases by leading the reader to assume that a visual “record” is further evidence that a story is truthful. The inclusion of an image to accompany a fake news story also increases the likelihood that the story will be shared on social media (Fenn et al., 2019). Echo chambers (Boutyline and Willer, 2017) and filter bubbles (Holone, 2016) created through social media platforms may further reinforce these cognitive processes, through the aforementioned heuristic of confirmation bias (Kahneman and Tversky, 1973). Research suggests that the acceptance of fake news items can be combatted through the application of epistemic cognition, which refers to how we gather knowledge about our world and develop our belief systems.

Our understanding of our social world is also influenced by what we perceive to be the beliefs and attitudes of our peers. As noted above, this can lead to the proliferation of fake news, but the same social influence can also be a powerful tool in combatting fake news. Indeed, it has been observed that viewing critical comments from friends that challenge fake news items on social media is more effective, and prompting people to question the item themselves than a disclaimer from the social media provider stating that item appears to be fake (Colliander, 2019).

Emotions are another determinant of the acceptance or rejection of fake news, as predicted under the feelings-as-information theory (Schwarz, 2012). Falsehoods are 70% more likely to be retweeted than the truth (Vosoughi et al., 2018). This could relate to several other forms of cognitive bias. Survival information bias refers to our tendency to pay attention to information that relates to the health and well-being of ourselves or those we care about (Stubbersfield et al., 2015). An example of this would be the tales of poison found within Halloween candy that are shared among parents each year, despite there being no record of this ever happening (Snopes, 2020). Such stories that invoke survival information bias in turn prompt emotional reactions. Similarly, social information bias refers to our tendency to attend to information that represents some form of deviation from social values or social norms (Stubbersfield et al., 2015). An example of the exploitation of this type of bias would be fake news stories, which are based on a politician or celebrity being involved in a conspiracy of some type. This again stimulates an emotional response, which as predicted by the feelings-as-information theory (Schwarz, 2012) may be accepted as truthful. In keeping with theories of social gossip, this information is then shared throughout the individual’s social network, as it has been identified by the individual as being of importance (Mesoudi et al., 2006). This continual reposting and endorsement of a fake news item throughout a social network may contribute to the phenomenon of illusory truth, the effect exploited by marketers in advertisements for many years in which statements that are repeated are seen as being more truthful (Dechene et al., 2010), a phenomenon observed in relation to fake news stories such as those relating to vaccines and autism (Unkelbach et al., 2019).

One argument made is that people will tend to assume that information they are exposed to is truthful, as they draw upon their experience of the base rate where most of the facts they encounter in their lives are mundane and accurate (Brashier and Marsh, 2020). This assumption may be attributed to an anchoring bias where the target believes that because they are honest, news sources are also honest. This is compounded when the information is received from a trusted source (Flanagin and Metzger, 2014).

Differences in cognitive ability appear to be another factor that predicts how easily a fake news story can be countered, with individuals who have low cognitive ability being less likely to change their initial acceptance of fake information when explicitly presented with the correct information (De Keersmaecker and Roets, 2017). This also relates to another cognitive bias known as the anchoring heuristic in which people will tend to keep any subsequent judgments close to their initial judgment, even if that initial judgment is proven to them to be incorrect (Northcraft and Neale, 1987). A Cognitive Reflection Test is used by Pennycook and Rand (2019) to show that susceptibility to fake news is dependent thinking rather than partisan bias; others support this rationale by citing increased vulnerability because of reduced analytical thinking and less open-mindedness (Bronstein et al., 2019).

#### Sociology

Cultural values and divisions caused by cultural values are used to define and continue the dialog. In many of the most recent cases, these values are used to stoke divisions and amplify societal polarizations (Azzimonti and Fernandes, 2018). Interestingly, many of the divisions within the targeted society are tribal in nature, indicating that certain base values are similar in both groups (i.e., a desire to be treated equally; a desire to freely express one’s views) but that the “in group” versus “out group” dynamic that defines tribes within a society is the targeted societal fissure. This is relevant because if the base values are the same, the targeted groups can be manipulated into thinking that the other group is the problem using many of the same techniques. An example of such behaviors may occur when one group within a society seeks equal rights, and another group perceives that in order for the first group to gain rights, they must lose or give up some of their own rights. While, logically this is not the case, the perception remains.

Higher technologic capabilities and interactions associate with greater vulnerability to information warfare campaigns (Szfranski, 1997). The information systems make possible the transmission of information at much greater speeds and volume, and interpretation of the information occurs with the human. Szfranski (1997) identified a strategic and tactical component that identifies identification (strategic) and restricting (tactical) disinformation. We argue that at the strategic level a fully integrated interdisciplinary response is required in order to accurately respond at the tactical level.

Orientation differs based on cultural values and heritage (Szfranski, 1997). Russia has been particularly active in stoking the immigration crisis in European Union countries (Volodymyr, 2016). By speaking to fears of “outsiders” invading countries, an increase in nationalism has arisen in host countries; specifically, Russia has created and supported right-wing narratives that speak to native citizens’ fears of loss of cultural values and general well-being (ibid). Culturally speaking, most Western democracies exhibit a coexistence of high and low uncertainty avoidance (UAI) values (Hofstede et al., 2010). Hofstede et al. (2010) noted an association between nationalism and high UAI cultural values. According to Hofstede et al. (2010), high UAI values reflect a fear or uneasiness with the unknown, whereas low UAI values associate with a curiosity and willingness to learn about the unknown. The stoking of these and other cultural fissures or differences, in an open society, is easier with social media due to the wide reach of the communications medium.

Successful campaigns impose false realities on the human targets (Szfranski, 1997). Open societies are vulnerable to alternative viewpoints, the willingness to accept various viewpoints (Hofstede et al., 2010; Nisbett, 2010; Minkov, 2013; Sample, 2015), while normally a strength and defense against weaponized information (Szfranski, 1997), these cultural norms have been used against these societies through the promotion of carefully crafted false narratives that are in many cases quite sophisticated, and in all cases customized to the values of the targeted audience (Volodymyr, 2016; Sample et al., 2018).

#### Political Science: Influencing Policies

Fake news, or the tactical use of manipulative communications in a political context, serves to deceive political actors in relation to their strategic intentions regarding a situation. That may involve efforts to distort political positions and options so as to move citizens to vote (or refrain from voting) on grounds that misalign their preferences and actions. It may seek to cloud decision spaces for voters or political authorities by introducing spurious issues that misdirect attention. It may seek to inflame relationships between groups in a population by amplifying differences. In this sense, maligned fake news campaigns may be thought of as activities that place additional stress on political systems, thereby undermining their capacities to govern. On the other hand, a similar fake news campaign might deceive populations or political authorities in a direction that increases perceptions of trust, legitimacy, or the performance of the political authorities or the underlying system of governance (Easton, 1975). These are the elements of a political system that might be subject to support or stress; too much stress, Easton noted, could lead to system break down and violence). These latter efforts to support a political system by celebrating its achievements are a common practice in non-democratic countries such as the People’s Republic of China where government employees are often called upon to promote the legitimacy of the CCP online, and an increasingly strict regime of censorship has prevented the emergence of critical commentary online (King et al., 2017; Jensen and Chen, forthcoming). Such efforts might artificially inflate the support for a system to serve the interests of an existing elite and order at the expense of efforts from the public to change the system. Either situation undermines democratic participation in a political system through distortions in the capacity to provide feedback and in its receipt by citizens and/or political authorities.

Political science treatments of “fake news” have focused on two distinct aspects. First, there is a question of whether foreign actors spreading fake news could have distorted the election results in 2016 in the United States. Second, scholars have studied the growing use of the term in recent time within political contexts, particularly its use as an epithet against journalists and perceived (other) political opponents. In terms of the interference question, the majority of the research suggests that it has had little impact on elections for two reasons. First, it is hard to distinguish Russian troll communications from other online sources, particularly those of the alt-right (Benkler et al., 2018). This delineation between Russia and alt-right publications is blurred since Russia today is a primary source of news stories for alt-right publications (Dorrell, 2017) that proceed to pass their article to mainstream conservative sites.

Related to this is the fact that domestic sources of news production and the campaigns themselves had considerably further reach than the Russian efforts, so it is unclear why Russian trolling would have produced an outsized effect compared to these other sources (Sides et al., 2018). Further, there is general doubt about the extent to which online manipulation campaigns have any effects based on research that shows political campaigning to general has neutral effects as competing campaigns cancel each other out (Kalla and Broockman, 2018).

Underlying the analysis in political science is a focus on information as the relevant unit of analysis in understanding the effects of fake news or manipulative campaigning. This focus owes its legacy to studies of voters and voter behavior, which have emphasized the role of campaigns and media outlets in transmitting information to voters who make up their minds (Downs, 1957; Ferejohn, 1990). Information efforts are considered independent of each other such that all information received, whether from a foreign state actor’s manipulation campaign or domestic news sources, is equivalent in their potential effects. Jamieson (2018) notes that this is not necessarily the case as Russia has appeared to sequence its messaging in relation to information, which was not necessarily public at the time, and the sequencing of Russian activity and other actions can have unique and amplifying effects.

Further, although the literature in political science tends to find little evidence of a net effect of campaigning on voter choices, there are a few categories of places where they do find effects that may amplify the effects of targeted foreign interference activities. First, there is evidence that such campaigns have effects where candidates have controversial positions, and there is a lot of investment in mobilizing voters supporting those positions (Kalla and Broockman, 2018). That would be consistent with the normalization effects that a coordinated covert influence campaign can have by making certain positions appear more reasonable through repetition (Kahneman, 2011). Second, there is evidence that messages that provide grounds for people to express fear and to take a limited and discrete action based on that message (e.g., like, retweet, vote once) can help create a persuasive narrative identity narrative for a voter (Jamieson, 2018).

Beyond that, there is a literature in political science focused on “misinformation.” Misinformation is often distinguished from disinformation in that the former is factually incorrect (a category overlapping with definitions of fake news), whereas the latter involves the intentional distribution of factually false claims for the purposes of inducing a political effect (Bennett and Livingston, 2018; Chadwick et al., 2018; Tucker et al., 2018). In relation to the misinformation literature, there is research into the efficacy of correctives (Nyhan and Reifler, 2010, 2015; Vraga and Bode, 2017). Some evidence suggests that false information can be corrected, but those effects tend to be limited to cases where an article of belief is not directly connected to one’s belief and identity structure (Garrett et al., 2013).

Finally, there is an area of study in political science on the use of the term, “fake news,” as a political epithet. Journalists and political opponents have been targeted with this term (Tandoc et al., 2018). Research shows that since the election of Donald Trump, there has been an upswing in the use of the term by politicians as an attack on others in places such as Australia, and the use of the term is usually amplified through reporting in mainstream news where it is not contested (Farhall et al., 2019). Propaganda historically has been understood by political scientists to not only provide a favorable narrative for one’s own side but also to demoralize the enemy and undermine their will to continue the fight (Lasswell, 1927). There are wider corrosive effects on politics that some ascribe to this current era where the ability to know truths is often put into question, with some suggesting that we live in a “post-truth” political era (Keane, 2018). The consequence of undermining trust in political authorities, expertise, and expert systems can have many systemic implications beyond the discrete consequence of swaying an election as it can make a political system on the whole ungovernable through its polarizing effects (Singer and Brooking, 2018). Fake news today may involve a combination of foreign and self-inflicted wounds, which erode the will of citizens to participate in democratic political life—precisely the condition Tocqueville feared would give rise to a form of despotism (Tocqueville, 2010).

While the fake news has played a prominent role in politics, the movement of fake news into health and science is especially troubling. Particularly with COVID-19, the ramifications are serious, and in many cases deadly. In one case a couple chose to take chloroquine based on empty speculation about the drug and died (Neuman, 2020). In this case, the scientific community called for research, and the story as well as the scientific process became politicized.

#### Cybersecurity

Cybersecurity, a relatively new domain of war (Lynn, 2010), was slow to react to the fake news phenomenon despite the fact that information systems, particularly social media, were widely deployed in the targeting, delivery, and dissemination of disinformation. Some of the reluctance to engage centered around the censorship versus freedom of speech argument, but another reason for the reluctance was financial. Social media sites such as Facebook have business models that are heavily reliant on advertising money. Interestingly, Taboola has been a common advertiser associated with fake news (Neilsen and Graves, 2017).

The argument continued, but a change occurred when the role of Facebook and other social media sites became known in the selling of personal information to Cambridge Analytica (Cadwalladr and Graham-Harrison, 2018; Risso, 2018), where the data were mined for use in political circles. The sharing of personal information crossed the privacy boundary that is one of the core tenant areas of cybersecurity (McCumber, 1991). The sale of user personal data (Cadwalladr and Graham-Harrison, 2018; Risso, 2018) and the customization of disinformation for the targeted users supported Szfranski (1997) assertion that civilians as well as military personnel will need protection from disinformation.

Szfranski (1997) observed that information systems are the primary means by which adversaries collect information on targets. Artificial intelligence (AI) algorithms that create and re-enforce filter bubbles (Sîrbu et al., 2019) can amplify the development of echo chambers, reducing the individual’s ability to find unbiased news. Furthermore, personas in the filter bubble in agreement with the target’s views gain credibility with that group. Filter bubbles are further discussed in *Data Science: Processing Large Volumes of Fake News* of this article.

The speed and reach of the internet enabled the rapid spread of disinformation, suggesting that any viable solution to counter the spread will require the automation and detection associated with cybersecurity to play a role in this endeavor (Cybenko et al., 2002; Horne and Adali, 2017; Sample et al., 2018). Furthermore, Russian campaigns have been characterized by the intensity of their use of information systems to promote narratives (Volodymyr, 2016; Payne, 2017; Jensen and Sample, 2019).

Volodymyr (2016) identified large-scale ongoing hybrid operations that relied heavily on Internet connectivity and social media outlet including Syria (Turkey), the European Union, and Ukraine. Since then NATO and liberal Western democracies have been added to the list for operations that originate in Russia. Other countries and groups have been encouraged by Russia’s success. The global nature of the problem that resulted in an unanticipated use of Internet technologies made digital deceptions such as fake news and deep fakes a problem that now belongs in part to cybersecurity.

The drivers for change within the cybersecurity field are not currently strong enough to lead to the development of solutions. Until nation states seek to apply larger-scale solutions and adopt policy changes that encourage the security of individual privacy rights, the cybersecurity industry has no incentive as a financially driven business area to address the problem. Indeed, commercial bodies have helped create this situation by repeatedly removing individual rights for privacy on the internet and creating a new norm, accepted by users, which they should trade their data for convenient services. A combination of commercial and legal drivers with strong law enforcement and government agency support has eroded anonymity on the internet. A strong incentive to change existing norms is likely to come from the application of targeted information operations on political, military, and critical national security personnel on a scale that forces policy changes. However, without such a driver, until this paradigm changes and there are legal or financial costs associated with the commercialization of user data on the Internet, the main drivers enabling online fake news will continue. Unfortunately, the commercialization and lobbying that have emerged in this area make it unlikely that the cybersecurity industry will be able to address this problem in the near term, except as a supporting role to respond to policy change. Efforts to integrate financial payment systems with social media and news outlets are likely to further exacerbate the problem. In the meantime, greater education of users and policy makers and focused attempts to secure users through anonymized and secure applications are probably the best short-term solutions available to cybersecurity practitioners.

#### Data Science: Processing Large Volumes of Fake News

The work performed by Cambridge Analytica (Risso, 2018) exemplifies the growth of data science in the fake news space. Originally used in marketing, another discipline that processes inputs from psychology, sociology, and the arts, the algorithms have been more recently applied to political goals. There are several different types of algorithms ranging from history-based heuristics, through trees and neural networks. Each of these algorithms can be manipulated through their data to either re-enforce preferences or to steer preferences into a new direction. Furthermore, the algorithm-created filter bubbles that re-enforce beliefs are amoral and probabilistic in nature; thus, the stories presented to the user are similar in tone and accuracy.

The ability to manipulate AI outputs extends beyond fake news propagation and into all aspects of AI and machine learning (ML). The example of Tay (Risley, 2016), the Microsoft chatbot that was trained to be helpful, but ultimately became abusive, illustrates the ability to manipulate AI and unintended direction through input manipulation of legitimate data. Weight and data manipulation fuels research into adversarial machine learning (AML) and malicious use of AI (MUAI), and many of the deceptions that other domains encountered occurred earlier in fake news.

The application of AI and ML in the creation, dissemination, and countering of fake news represents a growth area in data science, and as such, much has yet to be discovered. Deep fakes provide an example of this growth area. While data science can provide fascinating new insights to existing problems, the examples listed above remind us that the power of data science techniques can be used for benign, malevolent, or benevolent applications. A key point to remember in data science is the importance of the query being processed. The phrasing of a query sets the algorithm on a weighted path based on the data learned during the ML process. Depending on the data classifications, results can vary to the same query; this action is observable when two people enter the same query into a search engine and receive different results.

Despite the problems that AI and ML introduce, combatting fake news and other digital deceptions will be a job for AI/ML; Horne and Adali (2017) have already shown successes in using ML trained hosts to detect fake news. The accuracy will improve as the rules become more complete. Should the rules lack completeness, then fake news detection will be limited to detecting stories that fit a known pattern, signatures in cybersecurity parlance, and signatures have a low to non-existent detection rate with novel approaches. AI/ML are extremely proficient at detecting patterns, but the patterns must be familiar to the software, hence the need to create rules based on knowledge garnered from other disciplines.

#### Theater: News as Entertainment

“All the world is a stage” (Shakespeare, 2009), and fake news has a theatrical aspect. Theater can be thought of as a communal art form where the audience and performer share the roles of subject, spectator, and benefactor (Abaka, 2014). Similarly, fake news, particularly in an interactive mode of delivery (i.e., talk radio, live interactive TV news shows, social media, etc.), creates the same dynamic.

Theater, like fake news, is also interdisciplinary. The theater consists of eight major disciplines:

acting, directing, writing, producing, costumes, set, sound, and lighting design (Jones, 2004). Each of these disciplines is discussed along with their role in the creation and delivery of fake news in support of seeking a shared, desired intention within the story and the audience.

Theater requires the disciplines working together to create or manipulate the audiences’ thoughts and feelings. Fake news particularly uses propaganda delivered on trusted mediums in personal spaces such as talk radio in a car, TV in the home, and social media on cell phones and personal computers. All of these devices are trusted transmission sources for the user. The messaging deploying the use of previously described propaganda techniques is delivered in an environment that the user has already deemed trustworthy.

The story is most effectively told when the disciplines fuse together, creating a final product that is greater than the sum of the collective parts Jones (Hostetter and Hostetter, 2011). Actors use voice, facial expression, and body movement to convey the message that captures and unites the conscious and unconscious minds through shared presentation and decoding of words and symbols (Hostetter and Hostetter, 2011). Similarly, reporters and commentators use their voice, facial expressions, and body movement to both consciously and unconsciously deliver a message.

Actors prepare by not only memorizing lines, but also by drawing on emotional experiences that audiences can easily understand and find relatable, by using physical movement through expressions and body movement to convey emotions bringing to life the text of pathos. Once the message has been crafted, the delivery must seal the emotional hold.

Once the message has been successfully delivered and sealed, the re-enforcement can be taken care of through secondary actors known as trolls and bots. Trolls, paid personnel used to amplify a message can assume the role of unseen actors in support of maintain the target’s engagement. Bots are the automated counterpart of the troll, performing the same duties through the use of AI.

An actor seeks to tap into the audiences’ emotional memory (Stanislavski, 1989). Sight and sound are entryways into the imagination. Once an actor commands these two senses, they have a pathway into the imagination and can direct and manipulate the mind. Stanislavski (1989) considered sight and sound the two primary senses, and touch, smell, and taste secondary senses that can be triggered through the primary senses. Stanislavski (1989) noted that if an actor can appeal to only one of the senses, the remaining senses will also be available to the actor, all in support of influencing the audience’s emotional memory. One part of the director’s responsibility is to work with the actors to set the tone and intention for the play. The news director works with the news anchor to convey the tone and content of the story. The same news story compared across news stations can vary in length, tone, and detail (Sample et al., 2018). In the case of interactive news, much like in the theater, mood and emotions can be extracted from an audience.

Another important role that the theater director plays is in deciding what parts of the story remain and what parts are cut. Similarly, in the news media, the director/producer/editor determines which stories, or portions of the selected story, are presented to the audience. Recalling the earlier discussion on thinking, an event that is not sensed is not perceived. As with AI/ML, the flow of information is controlled by an entity in support of a specific intention.

The writer relies on words, language, rhythm, pacing, and musicality of language to be delivered by a skilled actor or anchor. All are well-crafted and integrated to achieve the intention of the story. The integrated whole is executed to play upon the values and beliefs of their audience. The use of memorable quotes or rhymes creates an indelible memory for the audience and the actor. Word choice for the story writer fits into manipulative linguistic choices, particularly those associated with pathos. Easily remembered phrases associated with fake news are effective methods of delivering a memorable message.

The producer is the person in charge of operations including hiring of directors, actors, and other personnel who support the vision. Casting of anchors and reports sends an enormous conscious and unconscious message to the audience. Attractive speakers benefit from the halo effect, while less attractive speakers are perceived as being more untrustworthy (Zebrowitz and Franklin, 2014). This finding suggests that a less attractive person would need to compensate for the untrustworthy impression, possibly by using intellect. The producer is responsible for all aspects of the production from vision, through operations and budgeting. In short, the producer is the production CEO possessing the holistic vision.

Costumes add a sensual authenticity to the production. Something as seemingly small as hair and make-up can have a significant effect on how the audience perceives the actor in theater or the reporter for news. Consider the appearances of various news hosts and the consistency of appearance on each of the major news stations. Suits for men are the costume with ties being carefully chosen. Women, while not forced to wear suits, also have clothing rules that allow for some flexibility of choice (Hillman, 2013). Within the range of professional clothing attire, a range exists for various articles of clothing and accessories (Crilli, 2014; Moeslein, 2019). A clear difference can be observed when viewing before and after pictures of liberal and conservative program hosts.

Set design provides the context or the visual environment. Color can set the emotional tone. For news, a simple calming blue background attracts attention, as does the color red (Hillyard and Münte, 1984), which suggests serious fact-based messaging. Red is a universal alerting color that people are trained to stop and focus their attention on (Kuniecki et al., 2015). Breaking news alerts appear on a red background. When delivering fake news, a set matches the color scheme and set design of the traditional mainstream media news station where the news anchors are seated but leaning forward. A well-thought-out set design seamlessly supports the reporter’s performance.

Audio is the presence or absence of background sounds such as music; voices and other sounds are also used to set the mood and manipulate emotions. Dark low-pitched music accompanied with dark lighting and a dark background suggests a sense of foreboding, preparing the audience for bad news.

Lighting is the remaining theatrical discipline. Lights and the shadows cast by lights allow for creating a perception. Shadows cast over an object suggest something to hide or untrustworthiness. Bright light suggests honesty and integrity. A harsh bright light suggests shining a light on a dark or dirty subject, exposing what was hidden.

All of these to point out the conscious effort of an entire production team to seek out the desired behavior, thinking, and feeling of a target audience. When executed with precision and art, the audience has no real defensive against it. Even the best of professionals in this field find themselves taken away and moved by the production. The actor must live it, feel it, and experience the depth of human nature supported by all the other disciplines to achieve the goal of a super objective set by producers, directors, and writers. How better to manipulate people into seeing things our way and buying in to joining our team.

For years, the delineation between news and entertainment has continued to blur (Edgerly, 2017). News reporters, while publicly claiming neutrality, can convey messaging through expressions, intonation, and movements, all methods of non-verbal communication that actors draw on. Reporters can convey joy, anger disgust, outrage, and other emotions without changing the text of the story. The goal of all news reporters, much like an actor, is to keep the audience’s attention. To that end, reporters, particularly television and other video reporters, like actors, wear make-up, perform in well-lit conditions, and engage with the camera. Through costume, make-up, set design, writing, direction, lighting, and sound, the reporter, much like the actor, sets out to elicit the audience’s emotional and physical response.

Cybenko et al. (2002) noted the mundane aspects of factual data. While linguistics offers a strong starting point and works well with textual data, the staging of events such as protests (Gu et al., 2017; Mueller, 2019, p. 29) requires inputs from theater. Theater, like all art, relies on sensory stimulation. Theater integrates sensory experiences relying on triggering sight and sound directly and touch, smell, and taste indirectly. These inputs are designed to trigger sensing from the target, to shape the perception.

## Lessons Learned for Countering Fake News

Because the problem is interdisciplinary in nature, any response that does not take into account the various disciplines can only partially succeed at best. This means that models must be superimposed and incorporated into the response. While an automated response favors data science and cybersecurity, this response will not fully succeed if linguistics, psychology, and sociology, along with the frameworks defined in those disciplines, are not fused into the solution.

Data science provides insights but requires carefully worded questions and subject knowledge in order to form the best, most comprehensive queries. Cybersecurity-informed solutions tend to be reactive, suggesting that queries posed by cybersecurity personnel may lack abstraction and will result in no new insights even when new problem sites are discovered. Furthermore, the overall reactive posture may result in software missing new styles and techniques in fake news creation and dissemination.

When combining data science and linguistics, computational linguistics offers some of the tools that can enable rapid detection of propaganda; however, natural language processors (NLPs) have shortcomings that may be unfamiliar to data scientists. NLPs used in computational linguistics are able to quickly synthesize large volumes of data presenting common themes through “bag of words” outputs and sentiment analysis. An explanation of problems in each area follows.

### NLP Problems

Natural language processors group together common words and group emergent patterns in written text. In order to do so efficiently, several things must happen that can result in misleading outputs for the data scientist who programs the software, including punctuation removal, case changing, word-stemming, and filler word removal.

• Punctuation removal is problematic because “!” and “?” and quotation marks are all a part of the sensationalism that elicits emotional responses from the reader (Shukrun-Nagar, 2009; Cohen et al., 2018).
• Case changing occurs when software evaluates words the ASCII representation for “News” is different than the one for “news.” In order to work around this problem, uppercase letters are converted to lowercase, and in most cases, no problems result. However, with emotion-driven fake news, words in all uppercase are present to stimulate the visual sense; by changing these words to all lowercase, an important textual clue is removed.
• Word-stemming results when NLPs convert adverbs to verbs, or convert verb tenses to the root word, so that “quickly” and “quicken” become “quick.” The problem is adverbs are words that are used to elicit an emotional response (pathos); by stemming these words, another textual clue is removed.
• Filler word removal is performed when NLPs remove words such as “the,” “and,” and “he” with the goal of avoiding filler words outweighing nouns and verbs. The problem is that the removal of pronouns also results in the removal of “us,” “we,” “they,” “them,” important words in the “us” versus “them” narrative oftentimes deployed in propaganda.

The problems listed above are easily solved when the programmer is aware of their existence, and most often, the programmer remains unaware. The problem is that the NLP packages are a part of a larger software development effort, and the aforementioned examples are not considered. The developers do not know what they do not know. In many cases, packages can be modified, or preprocessers can be written that quickly address these problems. Much of NLP work aids AI/ML rules, so knowing the correct query to make falls outside of the expertise of the programmer tasked with writing the software.

### Countering Content

Cybenko et al. (2002) noted the importance of detecting the attack before the narrative can affect the target’s behavior. Of course, this suggestion seems to work posteriori as a method to prevent repeat mistakes. One suggestion was to detect the preconditions that exist as a method to inoculate the target (ibid). Another would be to incorporate rules of propaganda into computational linguistics. The authors propose that three-point model of computational linguistics to address contextualization of data, as well as the descriptiveness, pattern-spread analysis to address the temporal aspect, and archival reputation analysis that adds temporal, context, and descriptive values for additional analysis.

#### Computational Linguistics—Contextualization and Descriptive Analysis

Fake news, by its very nature, consists of texts. Sometimes multimodal (employing, for example, audiovisual material), but overwhelmingly written compositions, designed to be transmitted (and retransmitted) across electronic/digital media to a carefully selected target audience. As communicative artifacts, they are open to analysis through linguistic tools, and as persuasive communications, they can be related to the body of work that has been done over centuries relating to rhetoric (the art of persuasive communication) in general, and propaganda in particular (see, *inter alia*, Ellul, 1965; Jowett and O’Donnell, 1986; Pratkanis and Aronson, 1992; Connelly and Welch, 2003).

Despite the list of problems enumerated above with NLPs, for those engaged in combatting fake news, the ability to employ computer-aided social network analysis offers great potential for mapping (and ideally countering) the spread on misinformation online, by quickly identifying the key vectors of fake news and tracking (in near-real-time) the flow of fake news across the Web (Hardaker and McGlashan, 2016; Chetty and Alathur, 2018). The use of Computational and Computer-Aided Corpus Linguistics (the identification of key textual features through comparison of a target text with a corpus or corpora of reference texts) offers a real possibility to create automated tools for the identification of fake news through its linguistic content and its removal from social media without human intervention and for automated generation of effective counter-texts (Pérez-Rosas et al., 2018; Marquardt, 2019; Pathak and Srihari, 2019).

The rules of propaganda are well understood, and the ability to modify software to enforce rules is attainable, particularly when ML is combined with statistical tools such as group comparisons and correlations to compare text. Many of the lexical expressions found in propaganda are synonyms, which can easily translate to neighboring words in ML classifiers (e.g., “step” and “leap”); thus, when phrases containing neighboring words are evaluated computationally, they will show that the distance between is statistically insignificant. Compared against the traditional phrases used in conversation where the word distance is statistically different, the ability to analyze and provide decision-making assistance is near real time.

The other areas listed above can be quickly evaluated using simple linguistic tools or even scripts that can strip out and quantify punctuation symbols before they are removed without record. Similarly, the stemming problem can also be addressed as a part of the preprocessing while preserving the important metadata. Computational linguistics works alongside data science and can provide much of the inputs needed to feed training data rules.

#### Pattern Spread—Temporal Analysis

In order for fake news to spread, stories must be artificially promoted using trolls and bots (Rosenblatt, 2018). The use of these aids leaves digital traces. Vosoughi et al. (2018) illustrated this phenomenon showing the extreme volume of stories that saturate the news cycle. When combined with the credibility of receiving these false narratives from trusted sources and the speed in which these stories spread, preemptive inoculation can become problematic. The OSoMe toolbox developed at Indiana University^4^ is another tool that illustrates the overwhelming spread of fake news. So that even the informed reader, when trying to gather information on alternative views, not only does not receive those stories due to AI algorithm weighting, but also the overwhelming volume of stories with the fake narrative (Gu et al., 2017; Sample et al., 2018; Jensen and Sample, 2019). Presently fake news spread patterns are rather distinct, but since they rely on automated software. they can be easily adjusted. In addition to providing a dominant narrative in volume, this flooding behavior also manipulates sentiment analysis, providing deceptive data to analysts.

Suggestions by Cybenko et al. (2002) included information trajectory modeling as a countering tactic. Information trajectory allows for comparisons against historic data and distance measuring from the historic data. This approach can be used to model pattern spread as well as linguistic differences and was proposed by Sample et al. (2018). However, unlike the linguistics component, there exists a lack of data for pattern spread of factual narratives, and factual narratives will have varying baselines depending on the nature of the story. For example, a natural disaster with many casualties will show a different pattern spread than a special interest story, which differs from a news story surrounding a celebrity. If the fake pattern is the area of focus, the parties responsible will alter the behavior to keep the filters from detecting the pattern; in cybersecurity, this is known as “fuzzing.” “Fuzzing” occurs when a character is changed in the signature string, allowing the new malicious data to slip in, undetected by the security filters.

#### Metadata Analysis of Archival Information—Time, Content, Context, and Reputations

Data science has been used and suggested for solutions to digital deception. This shows the enduring value of messenger credibility as discussed by Flanagin and Metzger (2014). Reputations can be discredited for an affordable cost, and reporters are human, so mistakes will happen. A gullible reporter who is repeatedly fooled may not have a very long career in his/her chosen field, but a reporter who consistently reports the facts, with or without theatrics, will have a pattern that is worthy of being considered credible. Currently, this process is performed by humans when they evaluate the credibility of a source, and as we previously discussed, the human decision-making process in this area is flawed and under attack.

Data science can go beyond sentiment analysis and some of the other techniques discussed earlier in this article. Every proposed countering technique will generate metadata, data about the collected data. This data are prime for fresh insights. Some of the metadata fields of interest would include, but not be limited to, reporter information, publisher information, time information, context content, and linguistic characteristics. A brief discussion follows.

• Reporter information: Reporter bias scores, average story word count, average linguistic characteristics associated with reporter including % nouns, verbs, adjectives and adverbs, publisher associations, credibility score, and sources used.
• Publisher information: Publication bias scores, average story word count, average linguistic characteristics, reporters used, credibility score, average story lifecycle, average time to report, sources used.

Time information is important because fake news typically has short lifecycles because once the deception is discovered, not everyone wishes to propagate it further; thus, time distance from original source, lifecycle, early source trajectory.

This archive of metadata with the small sample set of fields provides a starting point and is by no means complete. This starting point allows for groupings to answer known questions and unstructured analysis groupings to inspire new lines of thought and question. Furthermore, by collecting and processing metadata, the actual use space is smaller, resulting in a smaller and more efficient archive, which contains links to the larger complete archives.

#### The Role of Artificial Intelligence and Machine Learning

The need to understand the rules in order to create AI/ML rules otherwise signatures on steroids is discussed. Using ML software and satire as training data, Horne and Adali (2017) were successful in demonstrating software could successfully detect fake news in the form of satire. While the findings were encouraging the study looked for a specific type of fake news, satire.

AI is highly dependent on the classification schemes attained through ML, and both AI and ML are highly dependent on the accuracy and veracity of the training data used. The fact that in cybersecurity the poisoning of training data is an area of research should serve as a cautionary point. When rules are done well, AI and ML can easily outperform humans on many tasks; however, questions remain open on AI biases that may be intentionally or unintentionally inserted by the programmer (Bellovin, 2019). Biases have been observed in facial recognition software (Nagpal et al., 2019), resulting in unanticipated outputs. The importance of balancing the training data requirements results in having to balance inadequate data that yield false results, or overfitting provides accurate findings but with little to no abstraction. Striking this necessary balance requires an understanding of rules that lie outside the discipline of data science and cybersecurity, the two disciplines in the enforcement arena, and into the behavioral sciences space.

#### Game Theory to Inoculate—An Alternative Approach

Immunizations against diseases occur from patient exposure to a weakened form of the virus; this forms the basis for work done at Cambridge University (Van der Linden et al., 2017; Roozenbeek and Van Der Linden, 2019). While game theory was not discussed above, game theory does incorporate many of the aspects discussed in the previously mentioned disciplines that feed decision-making. The researchers found that by exposing participants to fake news stories. The researchers at Cambridge introduced the topic of attitudinal inoculation where subjects are warned preemptively, and false narratives are preemptively debunked, so that when exposed to deceptive data, they discard the information. Similar approaches work in cybersecurity when users are preemptively warned about spam and malware (Bindra, 2010). The research shows promise but requires prior knowledge of the false narrative before the narrative is created and distributed. In some controversial areas such as politics and climate, science may be difficult to anticipate the new narrative, or in cybersecurity terms, the zero-day narrative. In other areas, such as fake news surrounding medicine or economics news, the scope is more limited, making possible success in the exercise in anticipation.

## Examples Using the Model

The three-point model relies on evaluating using linguistic features, pattern spread, and archival reputation analysis. Included are some examples of the evaluations. On February 16, 2020, when the COVID-19 pandemic was in the early stages, Dr. Anthony Fauci was interviewed by CBS news on the show Face the Nation. A transcript of the show^5^ can be found in the public domain. Dr. Fauci’s remarks about the virus were approximately 646 words, containing no special punctuation, and the ratio of adverbs to text was 1:215. A few days later on February 25, 2020, Rush Limbaugh also shared thoughts on the same subject, the virus, during his talk show. Mr. Limbaugh’s text was also made publicly available^6^. Mr. Limbaugh’s word count was 756 words, containing six special punctuation instances, five “?” and one “!” along with an adverb-to-text ratio of 1:126. Additionally, Mr. Limbaugh’s text invokes rhetorical devices discussed earlier such as plain folk talk. Thus, based on these few characteristics, Limbaugh’s words would deviate farther from ground truth than would Fauci’s. This is not to say that Dr. Fauci’s remarks are considered ground truth, but that Dr. Fauci’s words are measurably more trustworthy than Mr. Limbaugh’s. Limbaugh’s text would suggest more than one standard deviation off of ground truth, and Fauci’s remarks would be considered less than one-half standard deviation from ground truth.

**FIGURE 1:**
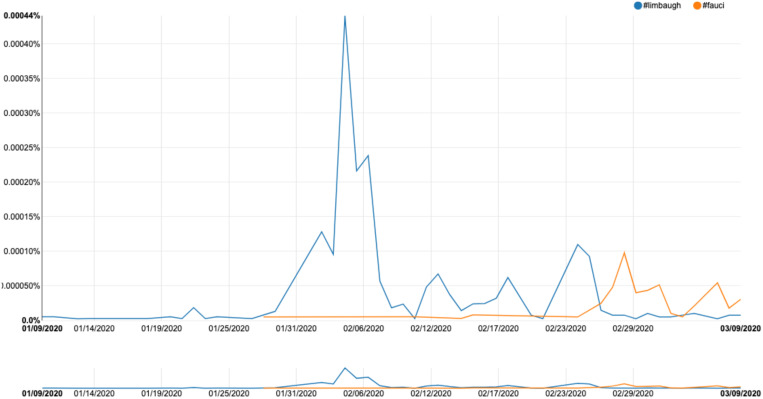
Trending data Limbaugh and Fauci mid-February.

This brings us to the pattern spread. The OSoMe tool^7^ allows for rapid tracking and visualization of hashtags and other social media features. When the Fauci and Limbaugh hashtags are compared in Figure 1, Limbaugh outperforms Fauci. Rush Limbaugh had been awarded the medal of Freedom in early February (notice the peak in the Limbaugh hashtag, followed by a drop that remains higher than Fauci’s even when Fauci’s interview is aired. Even more interesting is the delta between Limbaugh and Fauci on February 16, the date that the Fauci interview aired. The pattern spread appears to show the fact-based narrative underperforming. Only in March, once the pandemic had taken hold did Fauci surpass Limbaugh. Limbaugh had a full month (possibly longer) to deliver his version of the message to a larger audience.

**FIGURE 2:**
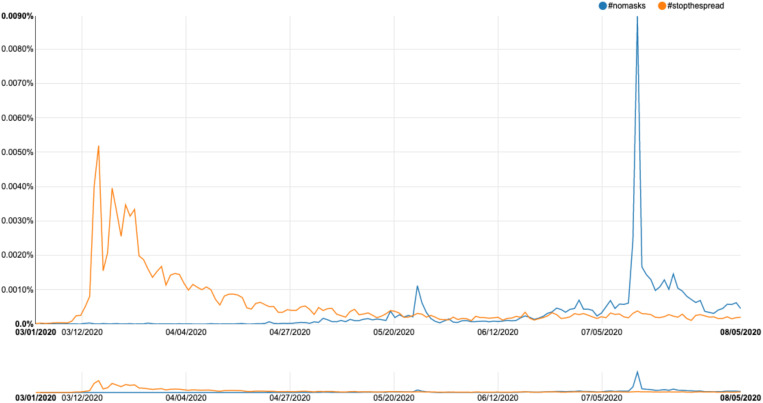
Hashtags from the mask debate.

**FIGURE 3:**
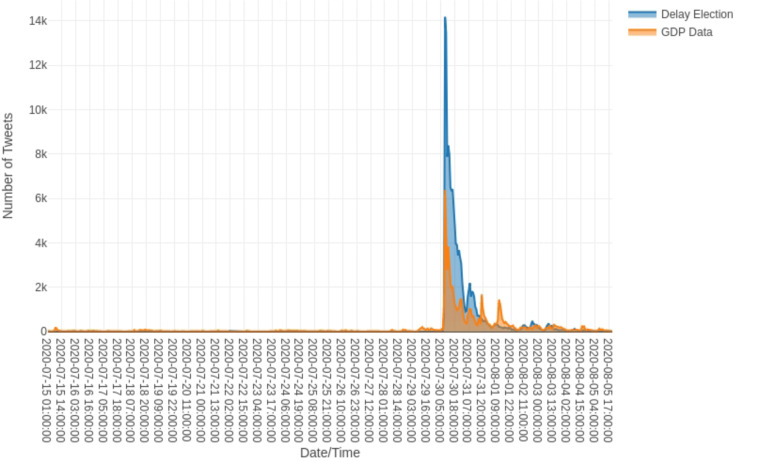
Twitter feeds for GDP drop and delay the election.

This same phenomenon can be observed again with the face mask debate where “#nomask” drowns out “stopthespread,” illustrated in Figure 2. Then more recently, this pattern is observed again with the “#movetheeletion” hashtag when compared to the real news of the sharp decline in GDP in the United States, as seen in Figure 3. The underperforming hashtags typically distribute longer, and the peaks tend to be shorter than their counterparts associated with fake narratives. Thus, the fake hashtags would result in the deviation value increasing. In the case of the #nomask hashtag, this pattern of a steep, dramatic peak with small tails was observed with election data in 2016 (Sample et al., 2018).

False or misleading narratives are also used to distract from factual news stories. This example was recently observed when President Trump suggested delaying the November election as the GDP quarterly data were released. In this case, the large drop in the US GDP would normally be the leading news as this drop would be associated with a significant economic recession. However, Figure 3 illustrates the Twitter feed of tweets for delaying the election that drowns out the GDP data tweets on July 30–31, 2020.

Pattern spread data can be valued by measurement off of known good news stories pattern spread.

The final model point of reputation analysis can be thought of as a consideration of the source. In the Fauci–Limbaugh example, the source of the Fauci interview is widely considered reputable. In this very simple example, Dr. Fauci has a history of speaking accurately, particularly when discussing medical matters; thus, his overall accuracy value would be considerably better than that of Mr. Limbaugh. This reputation analysis could be further enhanced by examining the sources that picked up both speeches for publication and those sources could also be evaluated. A final scoring metric could come from fact checking organization, where a point is also given for debunking a story narrative. The overall analysis when the three-point model is applied is that the closer to zero the total score (linguistic, pattern spread, and reputation values) lands, the more factually accurate the story.

## Summary

Szfranski (1997) argued that information warfare attacks would be enacted against both information systems and belief systems and that leaders and their supporting non-combatants would both be targeted. This is currently the case with fake news. Szfranski (1997) thought that open societies, such as Western democracies, would have better defenses than their autocratic counterparts, but the results on this front are decidedly mixed. The current iteration of fake news or propaganda operates in a jujitsu fashion, where a target’s strengths are used against itself, something that was unanticipated with the rise of digital propaganda.

There are many different reasons for the widespread success of this iteration. Key factors include the refined targeting techniques, the breadth of the Internet reach, the trust of social media platforms, the financial incentives for data management companies to sell information, and the use of open values systems found in open societies against those societies. Some open societies (i.e., Finland, Estonia, Latvia) have demonstrated resilience to fake news (Atkinson, 2018), suggesting a possible common set of values that have not been investigated. However, implementing defenses or countering tactics at speed requires an interdisciplinary approach. Meeting this challenge requires deep interactions that reflect a true exchange of ideas and implementation of models outside the traditional disciplines that support a fused response to this new generation of espionage (Younger, 2018).

Interdisciplinary work can help provide a more unified front to a currently fragmented, open-ended institution (Waisbord, 2018). Cybersecurity can aid in explaining how social bots spread misinformation; this can help journalists understand how misinformation spreads online and address increasing frustrations controlling fake news propagation (Schapals, 2018). Psychology sheds light on the phenomena of fake news amplification by means of echo chambers and confirmation bias to provide more conscious perspectives of how news, whether fake or not, is consumed in different cultures and groups. Akin to other areas, for journalism to adapt to change cultivated by the internet, a discussion should be initiated that recognizes the integration of multidimensional solutions. Journalists not only are responsible for the integrity and truth of their own reports, but also recognize that existing understanding of fake news spread is foggy (Mhamdi, 2016). Interdisciplinary approaches should acquit journalists from being solely responsible for policing fake news in today’s contemporary digital environment and emphasize collaboration to account for other trends responsible for fake news proliferation in our information streams.

## Author Contributions

CS: lead author, contributed to all sections, primary authorship on sociology, cybersecurity, data science, sections “Lessons Learned for Countering Fake News” and “Examples Using the Model.” MJ: sections “Introduction” and “Background,” primary authorship political science. KS: sections “Introduction” and “Background,” primary authorship linguistics. JM and AB: primary authors psychology. SF: primary author theater arts. DO and AO: secondary authors deception and cybersecurity. All authors contributed to the article and approved the submitted version.

## Conflict of Interest

The authors declare that the research was conducted in the absence of any commercial or financial relationships that could be construed as a potential conflict of interest. Two researchers were funded by the US Army Research Laboratory, as listed in section “Author Contributions.”

## Footnotes

1 This is not to say that identity-based logics cannot be subject to spatial modeling (see Glazer, 1995).

2 This is not to invoke a strong subject–object distinction denoting the object of description need not define the content and sources of subjects.

3 This is not to invoke a strong subject–object distinction denoting the object of description need not define the content and sources of subjects.

4 http://osome.iuni.inu.edu/

5 http://cbsnews.com/

6 http://mediamatters.org/

7 http://osome.iuni.iu.edu/
